# Prevalence and factors associated with hypertension among peoples living with HIV in East Africa, a systematic review and meta-analysis

**DOI:** 10.1186/s12879-023-08679-x

**Published:** 2023-10-25

**Authors:** Kirubel Dagnaw Tegegne, Getachew Asmare Adela, Gizachew Ambaw Kassie, Misganaw Asmamaw Mengstie, Mohammed Abdu Seid, Melkamu Aderajaw Zemene, Sefineh Fenta Feleke, Tadesse Asmamaw Dejenie, Endeshaw Chekol Abebe, Denekew Tenaw Anley, Anteneh Mengist Dessie, Molalign Melese Gesese, Nega Yimer, Natnael Atnafu Gebeyehu

**Affiliations:** 1 Department of Nursing, College of Medicine and Health Science, Wollo University, Dessie, Ethiopia; 2 School of Public Health, College of Health Science and Medicine, Wolaita Sodo University, Wolaita Sodo, Ethiopia; 3 Department of Epidemiology and Biostatistics, School of Public Health, Wolaita Sodo University, Wolaita Sodo, Ethiopia; 4 Department of Biochemistry, College of Health Science, Debre Tabor University, Debre Tabor, Ethiopia; 5 Unit of Physiology, Department of Biomedical Science, College of Health Science, Debre Tabor University, Debre Tabor, Ethiopia; 6 Department of Public Health, College of Health Sciences, Debre Tabor University, Debre Tabor, Ethiopia; 7 Department of Public Health, College of Health Sciences, Woldia University, Woldia, Ethiopia; 8 Department of Medical Biochemistry, College of Health Science, Gondar University, Gondar, Ethiopia; 9 School of Midwifery, College of Medicine and Health Sciences, Wolaita Sodo University, Sodo, Ethiopia

**Keywords:** Hypertension, Peoples living with HIV, Meta-analysis, East Africa

## Abstract

**Background:**

In recent years, improved access to effective antiretroviral therapy has meant that people living with human immune virus are living longer than before. The burden of non-communicable diseases particularly, hypertension parallels with the increase in age. Although hypertension screening is thought to be an effective indicator of overall health status and paves the way for early interventions in peoples living with human immune virus, the exact prevalence of hypertension in this population remained unknown. We aimed to report the prevalence of hypertension and examine the factors associated with hypertension among people living with human immune virus in East Africa.

**Methods:**

In this systematic review and meta-analysis, we searched PubMed, Science Direct, Scopus, Cochrane library, and Google Scholar databases for studies published until January 1, 2023. The search period was from January 10/2023, to February 10/ 2023. Random-effect models were used to calculate the pooled prevalence of hypertension. Subgroup analyses were conducted to explore potential heterogeneity. The Funnel plot and Egger’s test were used to assess publication bias.

**Result:**

A total of 15 studies with 10,916 individuals were included in the present meta-analysis. The pooled prevalence of hypertension among people living with human immune virus was19.75% (95% CI, 16.07%-23.42%)),). The prevalence of hypertension was not differed between studies conducted 2014- 2019 and, studies conducted 2020–2022. The prevalence of hypertension was lowest in Ethiopia (16.13%) and highest in Tanzania (26.76%). Alcohol consumption (Adjusted Odds Ratio (AOR): 3.39, 95% CI: 2.35–4.43), diabetes (AOR: 2.64, 95% CI: 1.89–3.39), longer duration of HIV (AOR: 1.72, 95% CI: 1.15–2.3), male sex (AOR: 1.62, 95% CI: 1.43–1.8), obesity (AOR: 2.89, 95% CI: 1.94–3.84), and older age (AOR: 2.25, 95% CI: 2.0–2.5), were the factors associated with the presence of hypertension in people living with human immune virus.

**Conclusion:**

Our study shows that one in five peoples living with human immune virus have hypertension causing symptoms and impairment, therefore requiring treatment. Designing effective health screening and hypertension management intervention programs helps to prevent the occurrence of hypertension and promotes peoples’ overall quality of life.

**Supplementary Information:**

The online version contains supplementary material available at 10.1186/s12879-023-08679-x.

## Background

According to the World Health Organization report in 2018, approximately 38 million people living with HIV (PLHIV) in the world, the largest number being in Africa, with 25.7 million [1]. Recognizing the sustained threat, Joint United Nations Program on HIV/AIDS( UNAIDS) set a target in 2020 with the goal of ending the epidemic by 2030 [2, 3]. The ambitious sustainable development goal (SDG-3) set a 95–95-95 target (95% of people living with HIV to be diagnosed, of whom 95% are on treatment, of whom 95% are virally suppressed) for 2030 [3]. Despite the slow decline in the incidence rate of HIV infection over the past two decades, with improved survival due to the introduction of Anti-retroviral therapy (ART), more people are living with HIV than ever before [4]. As a result, as the number of people living with HIV (PLHIV) who are aged are growing, the likelihood of being affected by non-communicable diseases (NCDs) including, hypertension increases [5].

Hypertension is estimated to affect 1.13 billion people worldwide, of which two-thirds live in low and middle-income countries [6]. Currently, it is also the most prevalent non-communicable disease among PLHIV, particularly in people aged 40 years and above [7–11]. Studies reported that about 24% of PLHIV have hypertension [12]. A systematic review of published articles between 2000 and 2017 revealed that hypertension prevalence among PLHIV ranges from 6 to 22% in Sub-Saharan Africa (SSA) [13]. Studies in East Africa have reported a prevalence of hypertension among PLHIV ranging from 7.98% [14] to 43.3% [15].

Factors contributing to hypertension in PLHIV are multifactorial, inconclusive, and include traditional contributors (e.g. older age, male sex, obesity, family history, smoking, and comorbidities) [16–21], and in some studies HIV-related factors such as immunodeficiency [19], longer duration with HIV or advanced HIV stage [16, 22] and ART [23] were mentioned. However, other studies have reported no association between hypertension risk and HIV-related factors [18, 20].

Evidences shows that East Africa is the second most affected region by HIV next to Southern Africa in the world [24]. Given that PLHIV are vulnerable to non-communicable diseases, estimating regional burden of hypertension in this population is invaluable. A previous study in SSA described the association of HIV and atherosclerotic cardiovascular diseases in a review [13],while meta-analysis of prevalence and factors of hypertension among PLHIV in East Africa remained unknown. Despite individual studies reported the prevalence of hypertension among PLHIV, there is no systematic review and meta-analysis conducted to provide a more comprehensive overview of the problem in the region. Therefore, this study aimed at reaching the overall prevalence of hypertension, and to figure out the factors associated with hypertension among PLHIV. The present meta-analysis leveraged single study reports from East African countries to formulate recommendations for clinical practice and future research.

## Methods

We prepared and presented this study according to Preferred Reporting Items for Systematic Reviews and Meta-Analyses (PRISMA) [25] (Additional file 1).

### Search strategy

We identified potentially eligible studies by systematically searching databases of PubMed, Science Direct, Scopus, Cochrane library and Google Scholar that are published until January 1, 2023. The keywords used during the search were: ((((((((((((((((prevalence) OR (magnitude)) OR (epidemiology)) AND (hypertension)) OR (high blood pressure)) OR (increased blood pressure)) OR (elevated blood pressure)) AND (Human immunodeficiency)) OR (HIV patients)) OR (HIV/AIDS)) OR (peoples living with HIV)) AND (determinants)) OR (factors)) OR (associated factors)) OR (predictors)) AND (adults)) AND (Ethiopia). Search terms were based on PICO principles to retrieve relevant articles through the databases mentioned above. The search period was from January 10/2023, to February 10/ 2023.

### Study selection

Two members of the study team (KDT and NAG) independently screened all titles and abstracts after the initial de-duplication. The inclusion criteria were (1) cross-sectional study, case control study, cohort study, (2) use of a validated measurement tool to assess hypertension, (3) published in English as full length article, (4) inclusion of point prevalence data for the outcome. We excluded researches with fewer than 100 participants (due to high selection bias), conference papers or abstracts, articles without full texts, and studies with data that could not be obtained from the corresponding authors.

### Outcome of the study

The primary outcome of interest was the prevalence of hypertension among PLHIV. Hypertension was measured as a persistent elevated blood pressure, Systolic blood pressure (SBP) ≥ 140 mmHg and/or Diastolic blood pressure (DBP) ≥ 90 mmHg, or reported uses of antihypertensive medication [26].

#### Data extraction

We extracted data from all included studies into a customized Microsoft Excel spreadsheet.

Two of the research team (KDT and NAG) independently extracted information regarding author names, study time, publication year, study location, median or mean age, prevalence of hypertension, odds ratio for factors of hypertension among PLHIV. Any disagreements between coders were resolved by the third author (GAA). We assessed all included articles for retraction or any erratum.

### Quality appraisal

Quality appraisal was conducted independently by two reviewers (KDT and NAG) using the appropriate JBI critical appraisal tool for prevalence studies [27]. When required, discrepancies were resolved through discussion led by the third author (GAA). The critical appraisal tool for prevalence studies produced a score between 0 and 8. The tool has eight parameters with yes, no, unclear, and not applicable responses. We summed the number of questions that were rated as ‘yes’ to create a total score. The checklist involve the following questions: Q1: Were the criteria for inclusion in the sample clearly defined?, Q2: Were the study subjects and the setting described in detail?, Q3: Was the exposure measured validly and reliably?, Q4: Were the standard criteria measured the outcome objectively?, Q5 Were confounding factors identified?, Q6: Were strategies to deal with confounding factors stated?, Q7: Were the outcomes measured validly and reliably?, and Q8: Was appropriate statistical analysis used? **(**Additional file 2).

### Statistical analysis

A random-effects model for meta-analysis was used because of assumed heterogeneity between the studies.* I* ^2^ index were used to evaluate heterogeneity between studies. The *I* ^2^ index refers to the truly observed variation ratio [28], and 25%, 50%, and 75% of the *I* ^2^ respectively indicate low, medium, and high heterogeneity [29]. We assessed Publication bias using visual inspection of funnel plot symmetry and, the Egger test. On Egger test, p-values of less than 0·05 signified evidence of publication bias among the included studies. We performed subgroup analyses according to studies conducted between (2014 and 2019) and those conducted between (2020 and 2022) and geographical regions. Besides, a leave-one-out sensitivity analysis was performed by iteratively removing 1 study at a time to examine the effects of a single study on the overall estimate [30]. The results are presented in a forest plot as a point estimate with 95% confidence intervals (95% CIs). We reported odds ratio (OR) describing the possible association between hypertension and PLHIV, if provided by the original study. Finally, we did meta-regression analyses to explore the potential sources of heterogeneity in our pooled estimates. All analyses were conducted in STATA 14.

## Results

### Study selection

**Fig. 1:**
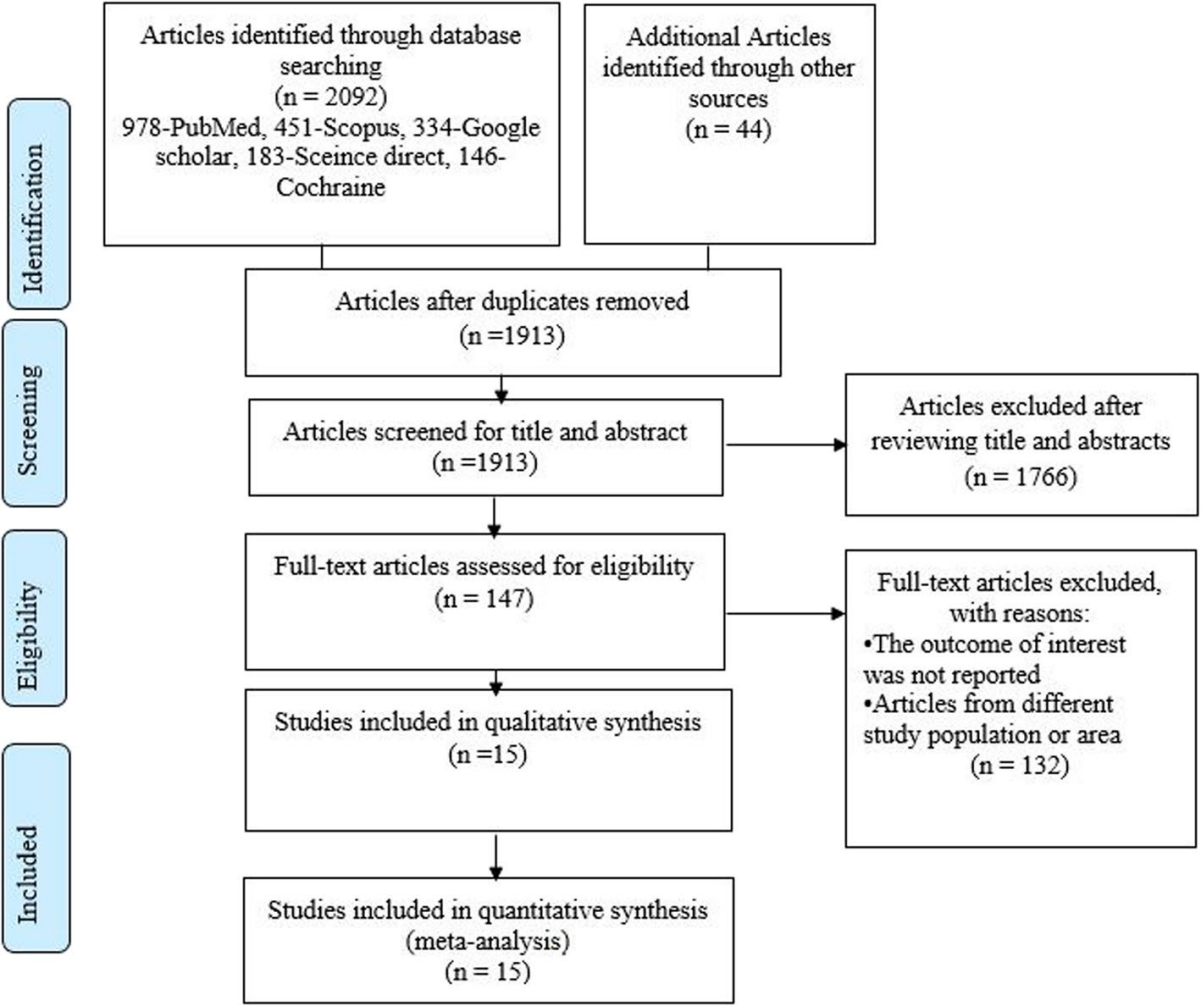
PRISMA flow chart illustrating the process of search and selection of studies included in the present systematic review and meta-analysis

Of the 2136 articles identified by applying search strategies, 1989 articles were excluded after a review of the title or abstract, leaving 147 articles assessed for full-text review, of which 15 met the eligibility criteria for this systematic review and meta-analysis. (Fig. 1).

### Studies characteristics

A total of 10,916 participants from all studies were included. The studies were published in the last decade, 2014–2022. The sample sizes ranged from 261 to 2026. Most studies (*n* = 13) used a cross-sectional study design [11, 14, 15, 31–40]. Two studies followed a retrospective cross-sectional design [41, 42]. Regarding countries where the study is conducted, 5 studies were in Ethiopia [31–34, 37], 4 in Tanzania [11, 15, 36, 38], 3 in Uganda [14, 41, 42], 2 in Kenya [39, 40] and 1 study in Burundi [35]. Twelve studies reported a response rate of 90% and above [31–42]. One study reported a response rate below 90% [11], and two did not report a response rate [14, 15]. All studies included participants with age above 18 years old. Thirteen studies reported participants included in terms of participants sex [11, 15, 31–36, 38–42]. Accordingly, the number of females was much higher than males in these studies. In most studies, the majority of PLHIV were on ART (*n* = 12, 80%). As a screening criteria for hypertension, all studies used a cutoff point of blood pressure “SBP ≥ 140 mmHg, DBP ≥ 90 mmHg. Sander LD et al. reported the lowest prevalence of hypertension, 7.98%, whereas the highest prevalence of hypertension was reported by Memiah P et al., 2021. Therefore, fifteen studies were evaluated, and the minimum quality score given is 5 out of quality scale which is 62.50%, indicating that they are low risk and included in the analysis (Table 1).

**Table 1:** Summary characteristics of studies included in the meta-analysis

Author	Pub.Year	Country	Study design	Sample size	Response rate (%)	Female to male (%)	Mean age	Prevalence	Quality
Ataro Z. et al. [31]	2018	Ethiopia	Cross-sectional	425	95.9	F = 69.9 M = 30.1	39.7	12.71	7
Gebrie A. et al. [33]	2020	Ethiopia	Cross-sectional	407	98	F = 60.4 M = 39.6	38.6	14.0	7
Getahun Z. et al [34]	2020	Ethiopia	Cross-sectional	560	97.7	F = 62.3 M = 37.7	39.3	14.11	7
Lukas K. et al. [37]	2021	Ethiopia	Cross-sectional	397	96.2	Not stated	35	11.08	6
Fiseha T. et al. [32]	2019	Ethiopia	Cross-sectional	408	96.6	F = 66.9 M = 33.1	37	29.66	7
Kagaruki GB. et al. [11]	2014	Tanzania	Cross-sectional	354	89	F = 70.5 M = 29.5	40.6	29.94	7
Kato I. et al. [36]	2020	Tanzania	Cross-sectional	612	100	F = 70 M = 30	41.2	16.01	6
Manavalan P. et al. [38]	2020	Tanzania	Cross-sectional	555	96.57	F = 78.6 M = 21.4	46	18.92	7
Memiah P. et al. [15]	2021	Tanzania	Retrospective cohort	261	Not stated	F = 76 M = 24	Not stated	43.3	5
Lubega G. et al. [42]	2021	Uganda	Retrospective cross-sectional	2026	93	F = 74.1 M = 25.9	Not stated	28.97	6
Kalyesubula R. et al. [41]	2016	Uganda	Retrospective cross-sectional	1996	99.8	F = 65.5 M = 34.5	31	20.94	7
Sander LD. et al. [14]	2015	Uganda	Cross-sectional	426	Not stated	Not stated	Not stated	7.98	5
Mbuthia GW. et al. [39]	2021	Kenya	Cross-sectional	939	93.9	F = 68.8 M = 31.2	44	18.85	7
Mogaka JN. et al. [40]	2022	Kenya	Cross-sectional	300	100	F = 50 M = 50	45	16.0	7
Harimenshi D. et al. [35]	2022	Burundi	Cross-sectional	1250	100	F = 81.6 M = 18.4	42.8	17.36	7

### Pooled prevalence of hypertension

**Fig. 2:**
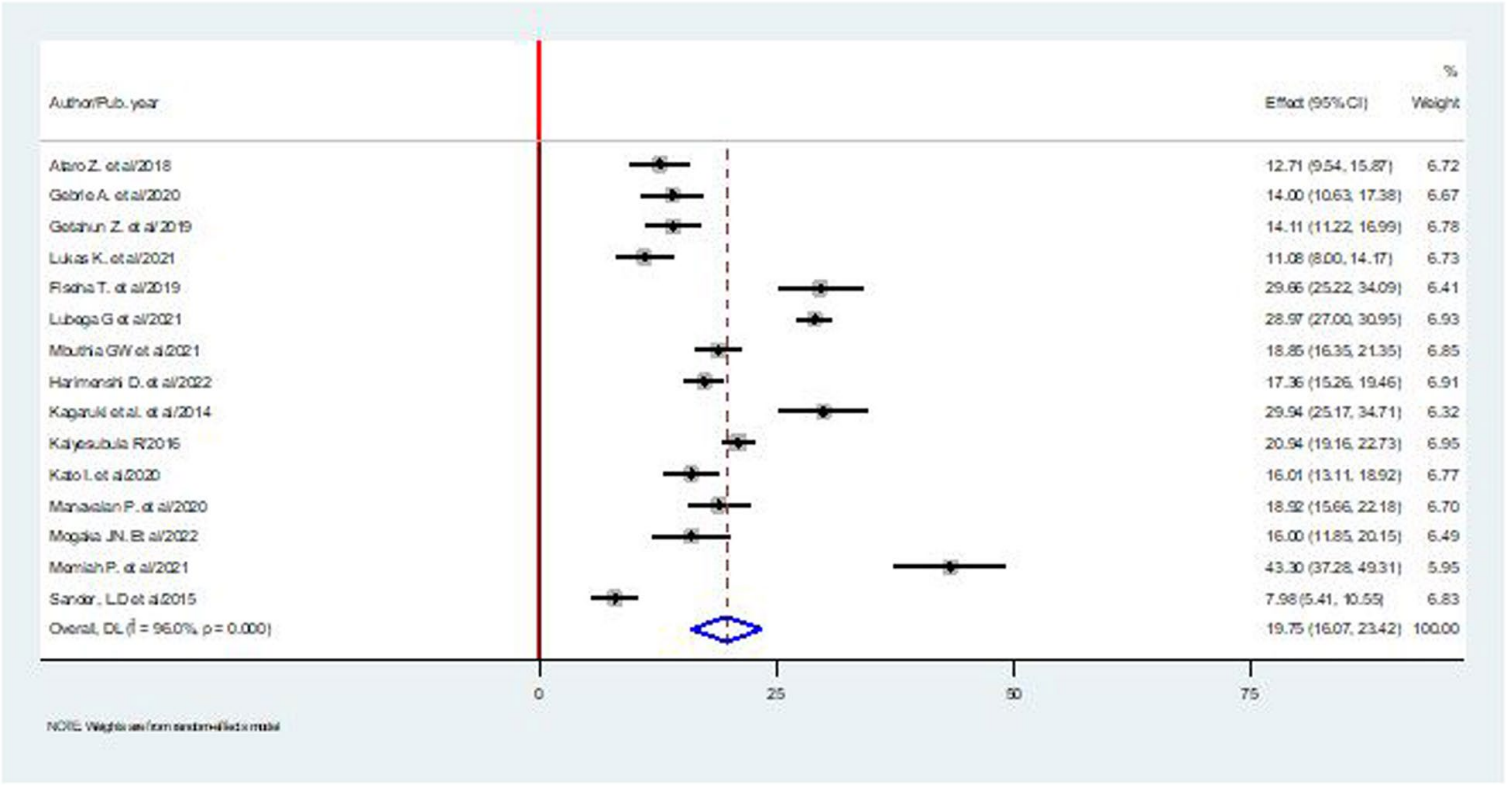
The pooled prevalence of hypertension among patients living with HIV in East Africa

Data from 15 studies with a total of 10,916 participants estimated the overall prevalence of hypertension among PLHIV to be 19.75% (95% CI, 16.07%-23.42%, I^2^ = 96.00%, *p* < 0.001) (Fig. 2). Sander LD. et al. reported the lowest prevalence, 7.98%, and the highest was reported by Memiah P. et al., 43.30%.

### Sub- group analysis

**Fig. 3:**
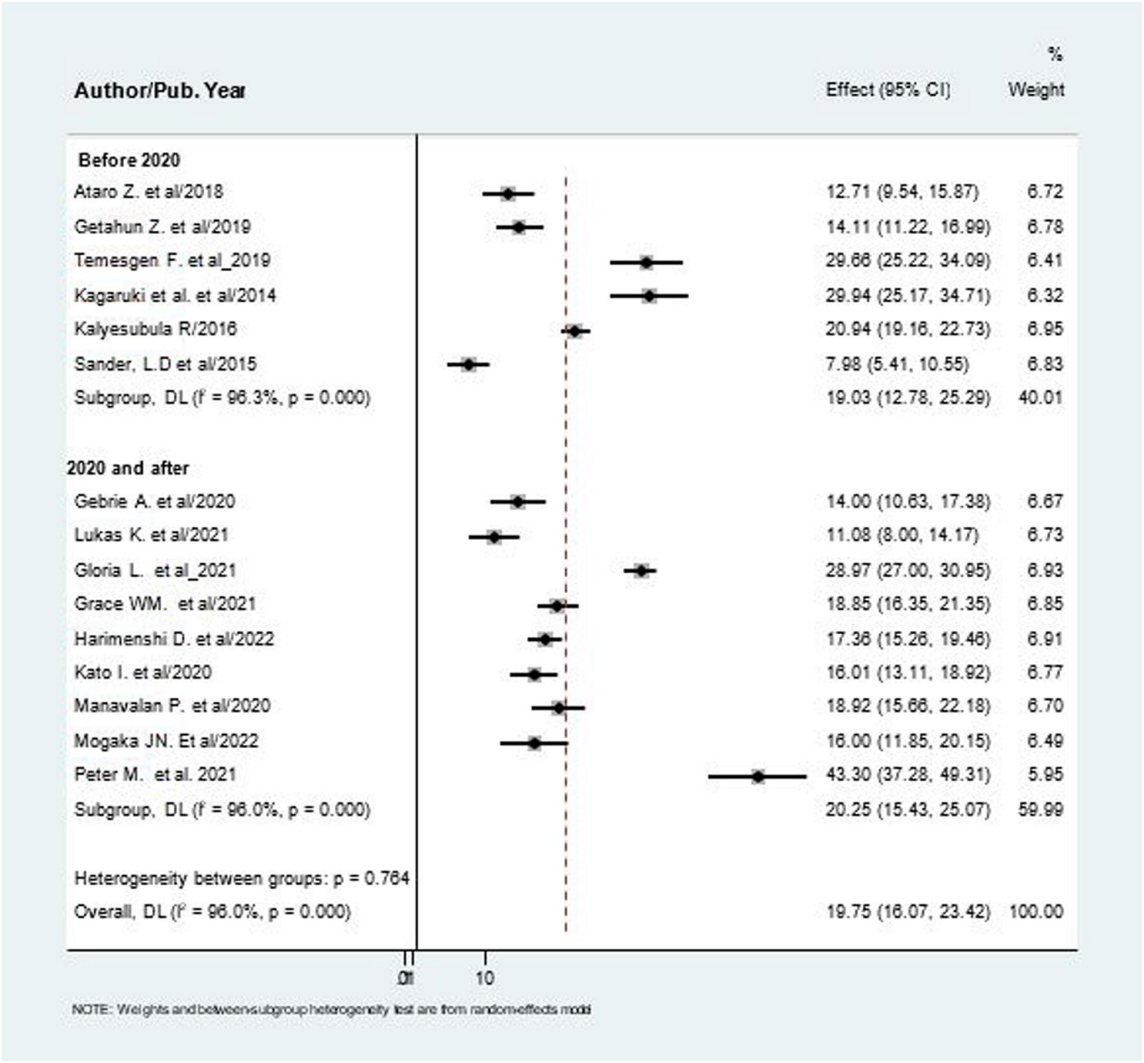
The pooled prevalence of hypertension among patients living with HIV based on publication year

**Fig. 4:**
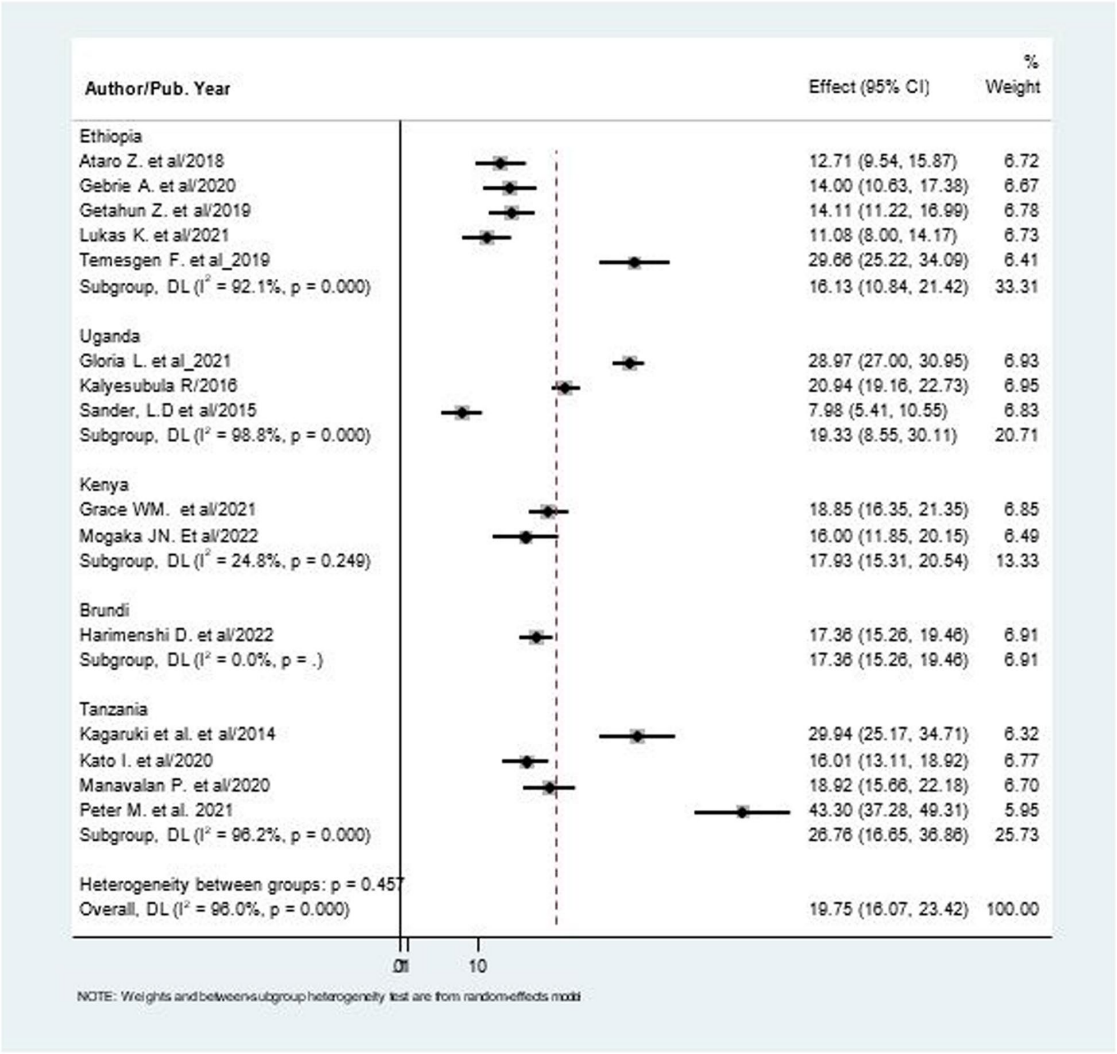
The pooled prevalence of hypertension among patients living with HIV based on study location

The pooled prevalence of hypertension among PLHIV was further compared by subgroups, namely whether the studies conducted between (2014 and 2019) and those conducted between (2020 and 2022), and by geographical regions. The prevalence of hypertension was not different in studies conducted between (2014 and 2019), and those conducted between (2020 and 2022). Regarding study location, the prevalence of hypertension was highest in Tanzania and lowest in Ethiopia (26.76% VS 16.13%). (Fig. 3, 4).

### Evaluation of publication bias

**Fig. 5:**
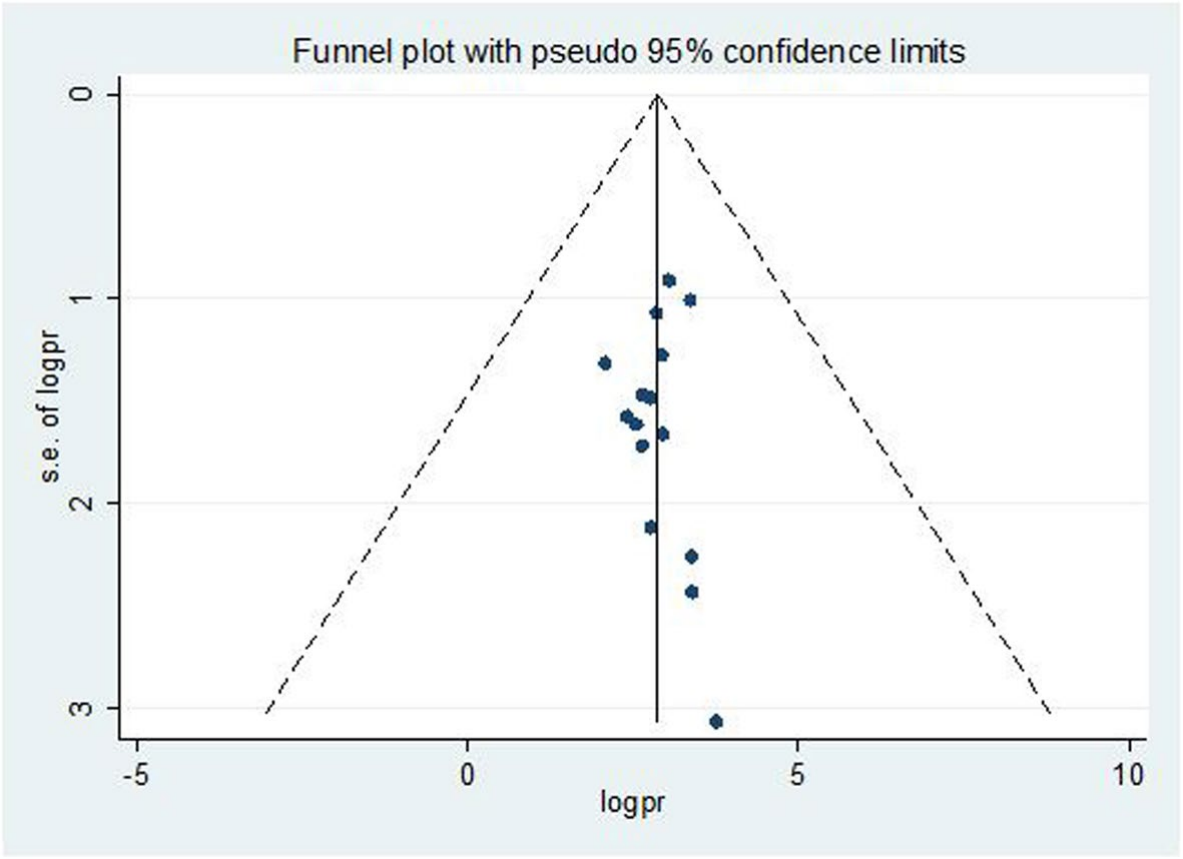
Funnel plot which shows the symmetric distribution of studies from the perpendicular line of no effect

We performed a subjective evaluation of publication bias through visual inspection of funnel plot and objective evaluation by running eggers test. The funnel plot shows a symmetrical distribution of studies from the line of effect (Fig. 5). Eggers test also showed that insignificant value for publication bias (*p* = 0.950).

### Meta regression and sensitivity analysis

Meta-regression was used to evaluate the impact of study characteristics on the pooled estimates. None of the study characteristics (publication year, sample size and study quality) was associated with the pooled estimates (*p* > 0.050) (Table 2).

**Table 2:** Meta-regression analysis of factors affecting between-study heterogeneity

Heterogeneity source	Coefficients	Std. Err	*p*-value
Publication year	0.032	0.16	0.200
Sample size	0.003	0.0005	0.500
Study quality	0.06	0.04	0.800

**Fig. 6:**
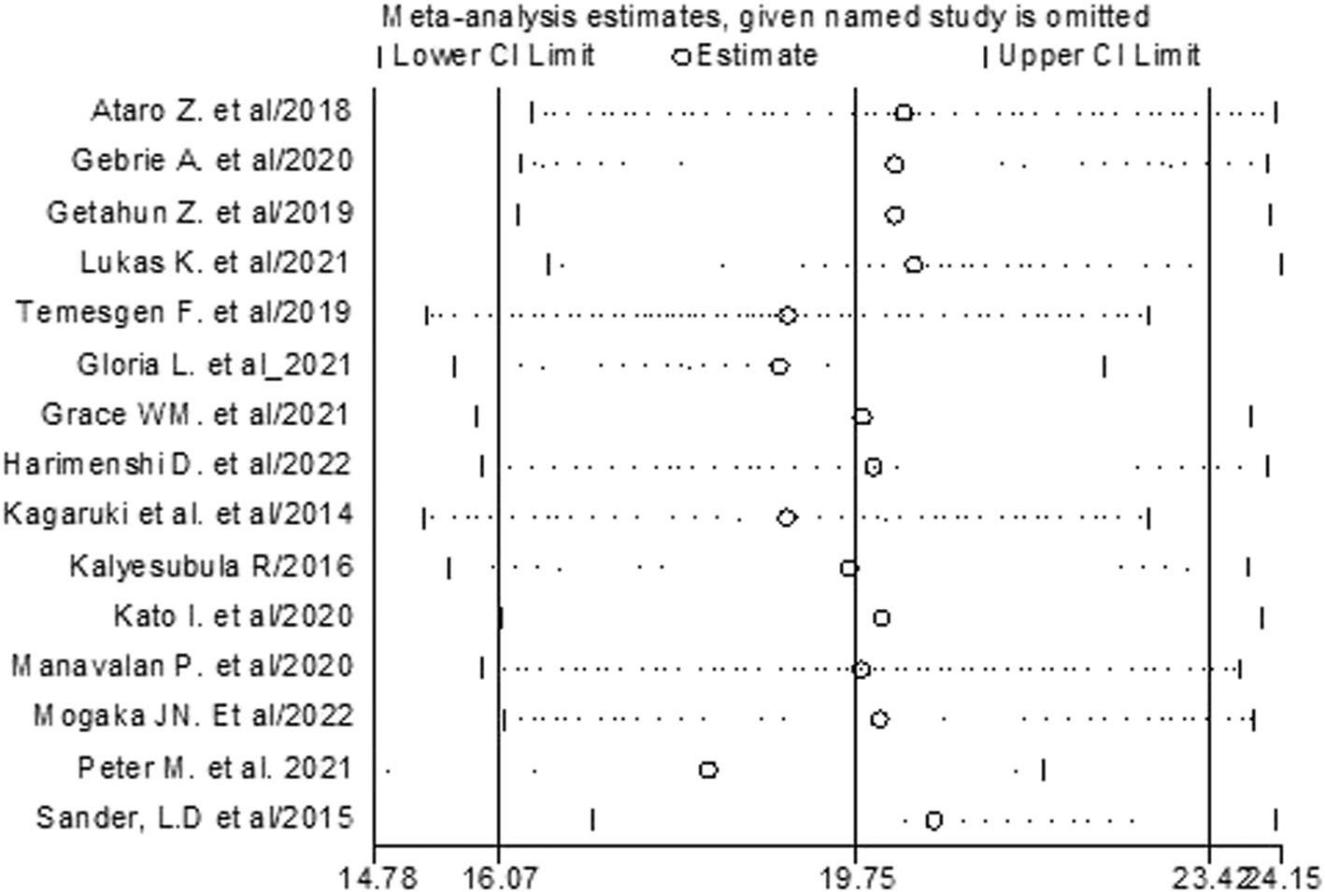
Results of sensitivity analysis of the 15 studies in the meta-analysis of hypertension among patients living with HIV

To assess the impact of an individual study on the pooled estimates, sensitivity analysis was conducted by excluding one study alternatively. Sensitivity analyses yielded no individual study influenced the pooled prevalence of hypertension among peoples living with HIV. When one study omitted from the analysis alternatively, the pooled prevalence of hypertension ranged from 18.28% to 20.50% (Fig. 6).

### Factors associated with hypertension among people living with HIV

Table 3 shows a total of 6 factors were included in the meta-analysis of hypertension among patients living with HIV. The identified factors were Alcohol consumption, Diabetes, longer duration of HIV, male, obesity, and older age. The forest plot of each associated factors was presented in Table 3.

**Table 3:** Summary estimates of odds ratio for factors of hypertension in PLHIV

Factors	Number of studies	Total sample (n)	Pooled OR (95% CI)	Heterogeneity I^2^ (%)	*p*-value
Alcohol consumption	3	1761	3.39 (2.35–4.43)^a^	82.4	0.003
Diabetes	3	2083	2.64 (1.89–3.39)^a^	66.8	0.049
Longer duration of HIV	5	2887	1.72 (1.15- 2.30)^a^	87.8	0.000
Male sex	3	2758	1.62 (1.43- 1.80)^a^	0.0	0.441
Obesity	8	4623	2.89 (1.94–3.84)^a^	96.0	0.000
Older age	8	6194	2.25 (2.00–2.50)^a^	97.8	0.000

*Note*: ^a^ indicates significant variables

### Alcohol consumption

The result is shown in Fig. 6. Three studies [31, 37, 39] with a total participant of 1761 explored this content. Random effect model showed that alcohol intake was significantly associated with hypertension in peoples living with HIV. Thus, peoples living with HIV who consume alcohol were 3.4 times more likely to have hypertension when compared to those who did not (OR: 3.39, 95% CI: 2.35–4.43, I^2^ = 82.4%, *p* = 0.003).

### Diabetes

Three studies [31, 32, 35] with a total participant of 2083 investigated this factor. The Random effect model showed that having diabetes is associated with hypertension in peoples living with HIV. We found that diabetes increased the odds of developing hypertension by 2.6 times as compared to without diabetes in PLHIV. (OR: 2.64, 95% CI: 1.89–3.39, I^2^ = 66.8%, *p* = 0.049).

### Longer duration of HIV

Five studies examined the associations of longer duration of HIV with hypertension in patients living with HIV. 4 [31–33, 37] reported a higher risk of hypertension for longer duration of HIV, and 1 [35] reported no associations. Compared to shorter duration of HIV, PLHIV with longer duration had a 72% enhanced risk for hypertension (OR: 1.72, 95% CI: 1.15–2.30, I^2^ = 87.8%, *p* = 0.000).

### Sex

Three studies examined the associations of being male with hypertension among PLHIV. Two reported increased risk of hypertension with being male, and 1 revealed no associations. Male living with HIV were 1.62 times more likely to develop hypertension (OR: 1.62, 95% CI: 1.43–1.80, I^2^ = 0.00%, *p* = 0.440).

### Obesity

Eight studies [11, 14, 15, 31, 32, 34, 35, 39] investigated the associations of obesity with hypertension among PLHIV. All reported obesity was associated with enhanced risk of hypertension. Likewise, obesity was increased the risk of hypertension 89% when compared to normal weight PLWHIV (OR: 2.89, 95% CI: 1.94–3.84, I^2^ = 96%, *p* = 0.000).

### Older age

Eight studies assessed the association of older age with hypertension among PLHIV. All studies revealed that older age was associated with hypertension in peoples living with HIV. Older PLHIV were 2.25 times more likely to have hypertension than younger ages (OR: 2.25, 95% CI: 2.00–2.50, I^2^ = 97.8%, *p* = 0.000).

## Discussion

Our systematic review identified 15 eligible studies reporting prevalence of hypertension, including 10,916 people living with HIV. By estimating the prevalence and identifying relevant factors on the prevalence of hypertension, this study can better help us identify PLHIV who are more likely to experience high blood pressure and develop relevant prevention strategies in a targeted manner. We identified alcohol consumption, diabetes, longer duration of HIV, male sex, obesity, and older age as an important factor associated with high blood pressure in PLHIV.

The overall prevalence of hypertension among PLHIV was 19.75%, which is consistent with a previous report of systematic review and meta-analysis in low and middle income countries (LMICs), 21.2% [43]. However, Our finding is lower than a global systematic review and meta-analysis of hypertension 23.6% in 2020 [12], and 25.2% in 2017 [44]. Narrow areal coverage of the review (East Africa) and limited number of individual studies in our study compared to the global systematic review could be the reason for the difference between the two regions. The high prevalence of hypertension among PLHIV implies the need for an integrated hypertension screening and continuous follow-up activities in HIV/ AIDS care services.

The subgroup analysis reported on basis of study location and publication year. Based on Country, Tanzania reported the highest prevalence of hypertension while the lowest was observed in Ethiopia (26.76% versus 16.13%). The highest heterogeneity observed in the overall meta-analysis (I^2^ = 96.0%) is somewhat explained by subgroup analysis by study location with no heterogeneity among studies conducted in Kenya (I^2^ = 24.8%) and Burundi (I^2^ = 0.00%). Subgroup analysis by publication year showed no significant difference between studies published between (2014 and 2019) and those published between (2020 and 2022). In areas where the prevalence of hypertension is higher among PLHIV, integrating hypertension services into routine HIV care by capitalizing the preexisting health system and following multidisciplinary approaches will be key [45]. It is also important to recognize that the trend in the prevalence of hypertension might vary over time in different countries as ART exposure, severity of the disease in PLHIV and access to healthcare service changes.

Our finding revealed the factors associated with the occurrence of hypertension among patients living with HIV. Thus, alcohol consumption, diabetes, longer duration of HIV, male sex, obesity, and older age were associated with the risk of hypertension. We found that alcohol consumption was associated with hypertension in peoples living with HIV. Several studies demonstrated the association of alcohol consumption and hypertension [46–49]. Multiple studies well documented Alcohol consumption risk factor for hypertension in multiple studies. Alcohol drinking results in a reduction of vasodilators like nitric oxide which leads to an injury of the blood vessel lining and eventually to hypertension [50]. Additionally, alcohol consumption creates accumulation of triglycerides and total cholesterol in the body, which ramp up the occurrence of hypertension [51]. Health care providers need to pay particular attention on any sign of alcohol drinking amongst PLHIV to prevent hypertension and promote a healthy lifestyle.

Diabetes was also associated with hypertension in PLHIV. A study in South Africa confirmed this report [46]. Diabetes is a well-known factor for hypertension in the general population. High blood sugar makes create formation of plaque that impede normal flow of blood which potentially increase blood pressure. Interventions and screening targeted for PLHIV with diabetes underscore a priority.

This study reported people with a longer duration of HIV were more likely to have hypertension than shorter duration. PLHIV with longer duration tend to be older than PLHIV with shorter duration and more likely to have hypertension in PLHIV. Studies reported that older age is the critical factor for development of hypertension [52, 53]. Several factors that might come with increased age such as, age related physiologic changes, comorbidities, and stress could contribute for occurrence of hypertension. Being male is the other factor for hypertension in peoples living with HIV. This finding is similar with studies conducted in Vietnam, Nepal and Ethiopia [53–55], whereas it was not similar to study conducted in Uganda [56]. The possible explanation is males are more vulnerable to behavioral risk factors such as alcohol drinking, cigarette smoking and other substances use which in-turn have direct link with development of hypertension.

Advanced age has been identified as an important factor for high blood pressure in PLHIV. Our meta-analysis confirmed that older age was also correlated with occurrence of hypertension in PLHIV. To date, numerous studies pointed out older age is the critical factor associated with hypertension in the general population [53, 56–58]. Advanced age is one of the non-modifiable factor that affects the circulatory system of the body. Aging causes structural and functional changes in the blood vessels, impedes the normal flow of blood and subsequently will lead to increased blood pressure [59].

Many studies have reported that obese PLHIV were at risk of developing hypertension than normal weight. Our meta-analysis results also showed that obesity is the factor associated with hypertension in East Africa. Studies have shown that the relative risk of hypertension among obese peoples is 2.93. Foreign studies have also shown that obesity is highly correlated with hypertension in way that excessive distribution of visceral fat changes the level of hormones, induce inflammation, and damages endothelial cells. This variations in result in change in sympathetic nervous system, renal function, and micro vascular levels, which contribute to hypertension [60, 61]. Therefore, it is essential to advocate early weight control and introduce interventions on obese peoples.

Our result could be considered as a key as it informs policy implications for the management of hypertension and HIV. They highlight the need of strengthening health system nationally to provide effective prevention and care for hypertension, to mitigate long-term health impacts in the cardiovascular and renal complications [62]. Yet, multiple studies identified important gaps in the treatment pathway with a significant portion of patients remained either undiagnosed, untreated or hypertension is uncontrolled [63, 64].

This systematic review has strengths and weaknesses. We have used a robust searching algorithms to pull studies from multiple databases. This study provided hypertension prevalence estimates in East Africa, which makes the first study to date. In addition we identified factors of hypertension in PLHIV, which is very important for preventative public health. The limitations of this review include the following: First, only articles published in the English language were included, so we might miss a few articles. Second, High heterogeneity between studies in our meta-analysis implies that the prevalence of hypertension varies across studies. To overcome this constraint, we used a random-effects model and performed subgroup analyses. Furthermore, considerable heterogeneity may indicate that the prevalence of hypertension varies significantly by geographical region, country, gender, age group, or study methodology. Third, as the included individual studies are cross-sectional design in nature, it may have some weaknesses to establish a causal association between hypertension and the observed independent factors. Finally, some factors were only reported by one study, thus we were not able to generate the pooled effect size. We suggest that future prospective studies should be conducted in PLHIV to estimate the prevalence of hypertension and figure out associated factors.

## Conclusion

This systematic review provides the first comprehensive overview of hypertension among PLHIV in East Africa. Based on available evidence, we found that the prevalence of hypertension among PLHIV is high in East Africa. Alcohol consumption, diabetes, longer duration of HIV, male sex, obesity, and older age were potentially linked with presence of hypertension in PLHIV. There is a need for development of public health strategies for prevention and early management of hypertension among PLHIV. Our findings justify that further research is required in terms of identification, treatment and support with management of hypertension among PLHIV in the region.

### Supplementary Information

**Additional file 1.** PRISMA checklist.

**Additional file 2.** Methodological quality assessment of included studies using Joanna Brigg's Institute quality appraisal criteria scale (JBI). The eight-item questions assessing inclusion criteria, study setting and participant, exposure measurement, objectives, confounder, statically analysis, outcome measurement, and dealing confounder were used.

## Data Availability

All relevant data are within the Manuscript and its Supporting Information files.

## Abbreviations

ART: Anti-retroviral Therapy
PLHIV: Patients Living with HIV
JBI: Joanna Briggs Institute
LMICs: Lower and Middle Income Countries
PRISMA: Preferred Reporting Items for Systematic Reviews and Meta-Analyses
WHO: World Health Organization
