# Supplementary material for: Prevalence and factors associated with hypertension among peoples living with HIV in East Africa, a systematic review and meta-analysis
Source: BMC Infect Dis. 2023 Oct 25;23:724. doi: 10.1186/s12879-023-08679-x (PMC10601241; doi:10.1186/s12879-023-08679-x)
Supplement: Supplementary file 2 — Additional file 2. Methodological quality assessment of included studies using Joanna Brigg's Institute quality appraisal criteria scale (JBI). The eight-item questions assessing inclusion criteria, study setting and participant, exposure measurement, objectives, confounder, statically analysis, outcome measurement, and dealing confounder were used. [file 12879_2023_8679_MOESM2_ESM.docx]

S 2 Table. Quality assessment for the included Studies

| Item | The criteria for inclusion in the sample clearly defined | Describe study setting and participant | Valid and reliable exposure measurement | Objective and standard criteria for measurement | Identified confounder | Strategies to deal with confounders | Valid and reliable outcome measurement | Appropriate statically analysis | No of ‘yes’ ‘ |
| --- | --- | --- | --- | --- | --- | --- | --- | --- | --- |
| Ataro Z. et al | Yes | Yes | No | Yes | Yes | Yes | Yes | Yes | 7/8=87.5 |
| Gebrie A. et al | Yes | Yes | Yes | Yes | Yes | No | Yes | Yes | 7/8=87.5 |
| Getahun Z. et al | Yes | Yes | No | Yes | Yes | Yes | Yes | Yes | 7/8=8.75 |
| Lukas K. et al | Yes | No | No | Yes | Yes | Yes | Yes | Yes | 6/8=75 |
| Fiseha T. et al | Yes | Yes | No | Yes | Yes | Yes | Yes | Yes | 7/8=87.5 |
| Kagaruki GB. et al | Yes | Yes | Yes | Yes | Yes | No | Yes | Yes | 7/8=87.5 |
| Kato I. et al | Yes | Yes | Yes | Yes | No | No | Yes | Yes | 6/8=75 |
| Manavalan P. et al | Yes | Yes | No | Yes | Yes | Yes | Yes | Yes | 7/8=87.5 |

| Memiah P. et al | Yes | No | Yes | No | Yes | Yes | Yes | No | 5/8=62.5 |
| --- | --- | --- | --- | --- | --- | --- | --- | --- | --- |
| Lubega G. et al | Yes | yes | No | No | Yes | Yes | Yes | Yes | 6/8=75 |
| Kalyesubula R. et al | Yes | Yes | Yes | No | Yes | Yes | Yes | Yes | 7/8=87.5 |
| Sander LD. et al | Yes | No | Yes | No | Yes | Yes | No | Yes | 5/8=62.5 |
| Mbuthia GW. et al | Yes | Yes | Yes | Yes | Yes | Yes | No | Yes | 7/8=87.5 |
| Mogaka JN. et al | Yes | Yes | No | Yes | Yes | Yes | Yes | Yes | 7/8=87.5 |
| Harimenshi D. et al | Yes | Yes | Yes | Yes | Yes | No | Yes | Yes | 7 /8=87.5 |
